# Chronic exposure to the star polycation (SPc) nanocarrier in the larval stage adversely impairs life history traits in *Drosophila melanogaster*

**DOI:** 10.1186/s12951-022-01705-1

**Published:** 2022-12-08

**Authors:** Shuo Yan, Na Li, Yuankang Guo, Yao Chen, Chendong Ji, Meizhen Yin, Jie Shen, Junzheng Zhang

**Affiliations:** 1 Department of Plant Biosecurity and MARA Key Laboratory of Surveillance and Management for Plant Quarantine Pests, College of Plant Protection, China Agricultural University, Beijing, 100193 China; 2 State Key Lab of Chemical Resource Engineering, Beijing Lab of Biomedical Materials, Beijing University of Chemical Technology, Beijing, China

**Keywords:** Nanoparticle, Biotoxicity, Star polycation, *Drosophila melanogaster*

## Abstract

**Background:**

Nanomaterials are widely used as pesticide adjuvants to increase pesticide efficiency and minimize environmental pollution. But it is increasingly recognized that nanocarrier is a double-edged sword, as nanoparticles are emerging as new environmental pollutants. This study aimed to determine the biotoxicity of a widely applied star polycation (SPc) nanocarrier using *Drosophila melanogaster*, the fruit fly, as an in vivo model.

**Results:**

The lethal concentration 50 (LC_50_) value of SPc was identified as 2.14 g/L toward third-instar larvae and 26.33 g/L for adults. Chronic exposure to a sub lethal concentration of SPc (1 g/L) in the larval stage showed long-lasting adverse effects on key life history traits. Exposure to SPc at larval stage adversely impacted the lifespan, fertility, climbing ability as well as stresses resistance of emerged adults. RNA-sequencing analysis found that SPc resulted in aberrant expression of genes involved in metabolism, innate immunity, stress response and hormone production in the larvae. Orally administrated SPc nanoparticles were mainly accumulated in intestine cells, while systemic responses were observed.

**Conclusions:**

These findings indicate that SPc nanoparticles are hazardous to fruit flies at multiple levels, which could help us to develop guidelines for further large-scale application.

**Supplementary Information:**

The online version contains supplementary material available at 10.1186/s12951-022-01705-1.

## Background

As adjuvants, nanomaterials with the ability to encapsulate and deliver pesticides are expected to preserve global food production without causing collateral environmental damage [1–4]. Widely applied in the agricultural system, exposure of wild life and human to nanomaterials are inevitable. Nanoparticles, by their nature, possess the ability to be transported into cells and important intracellular organelles, which may cause significant impacts once taken up by living organisms [2, 5]. Therefore, the safety of newly engineered nanomaterials should be comprehensively examined and evaluated before large-scale application [6, 7].

Various types of dimethylaminoethyl methacrylate (DMAEMA) polymers have been developed as nanocarriers for efficient drug and genetic material delivery, but their potential side effects have mostly been examined in cell lines so far [8–11]. Therefore, in vivo studies are required to dissect the hazards of DMAEMA polymers in living organisms. Our group has designed and synthesized a star polycation (SPc) nanocarrier with polymerized DMAEMA side chains [12]. SPc shows great potential as a nanocarrier for efficient delivery of various genetic materials and pesticides [13–18]. We have shown that at working concentrations, SPc showed no acute toxicity for soybean aphid [19], green peach aphid [16, 20] as well as a widely used predatory ladybird [21]. However, the impacts of SPc on animal development and health, especially the underlying molecular mechanisms are not fully understood. Examination of the biotoxicity of SPc would also help to understand the potential hazards of other DMAEMA based nanoparticles.

*Drosophila melanogaster*, the fruit fly, has become a commonly used animal model to determine the potential harmful effects of different types of nanoparticles [22–28]. The use of *Drosophila* as model animal benefits from the advantages such as small body size, rapid lifecycle, low cost and clear genomic information [29]. When used in nanotoxicity studies, the fly model can mimic the main entry routes of nanoparticles into animal body through oral administration [30] as well as inhalation [31].

The present study aims to examine the in vivo impact of SPc on *Drosophila* larvae development and adult health. The effects of chronic exposure to a sub lethal concentration of SPc in the larval stage were examined. Upon exposure to SPc at larval stage, the emerged adults were morphologically normal but their key life story traits were adversely impacted. This observation indicates that SPc nanoparticles have long lasting effects well beyond exposure times. Thus, further attempts were made to understand the mechanism of biotoxicity through transcriptomic analysis and examination of tissue specific responses in the larvae.

## Results

### Preparation and characterization of SPc nanoparticles

**Fig. 1:**
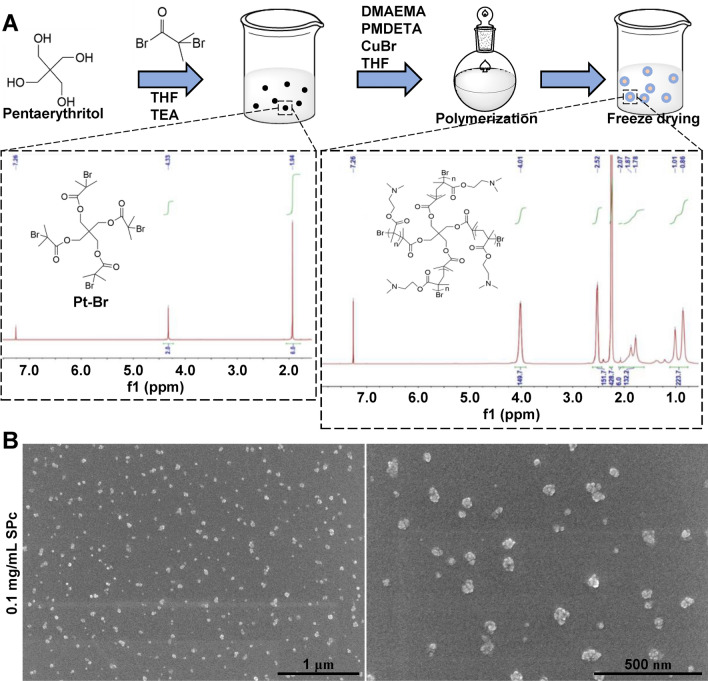
Preparation and characterization of SPc nanoparticles. **A** SPc was synthesized by first constructing the star initiator Pt-Br, which was then polymerized with DMAEMA. SPc was purified by dialysis and the final product was obtained as white powder after being freeze-dried. **B** SEM images of SPc nanoparticles

The SPc nanoparticles used in this study were newly synthesized and purified (Fig. 1A). When examined under scanning electron microscope (SEM), SPc particles were found to be spherical in shape (Fig. 1B and Additional file 1: Fig. S1). For the SPc nanoparticles used in the subsequent experiments, the average particle size was 46.84 ± 1.35 nm and average zeta potential was 20.47 ± 0.49 mV (Table 1).

**Table 1:** Particle size and zeta potential of SPc at the concentration of 0.1 mg/mL

Sample number	Particle size	Average particle size	Zeta potential	Average zeta potential
1	45.06 nm	46.84 ± 1.35 nm	19.50 mV	20.47 ± 0.49 mV
2	49.48 nm		20.94 mV	
3	45.98 nm		20.97 mV	

The particle size was calculated based on 100 particles from one SEM photo.

### Acute toxicity of SPc on fly larvae and adults

**Fig. 2:**
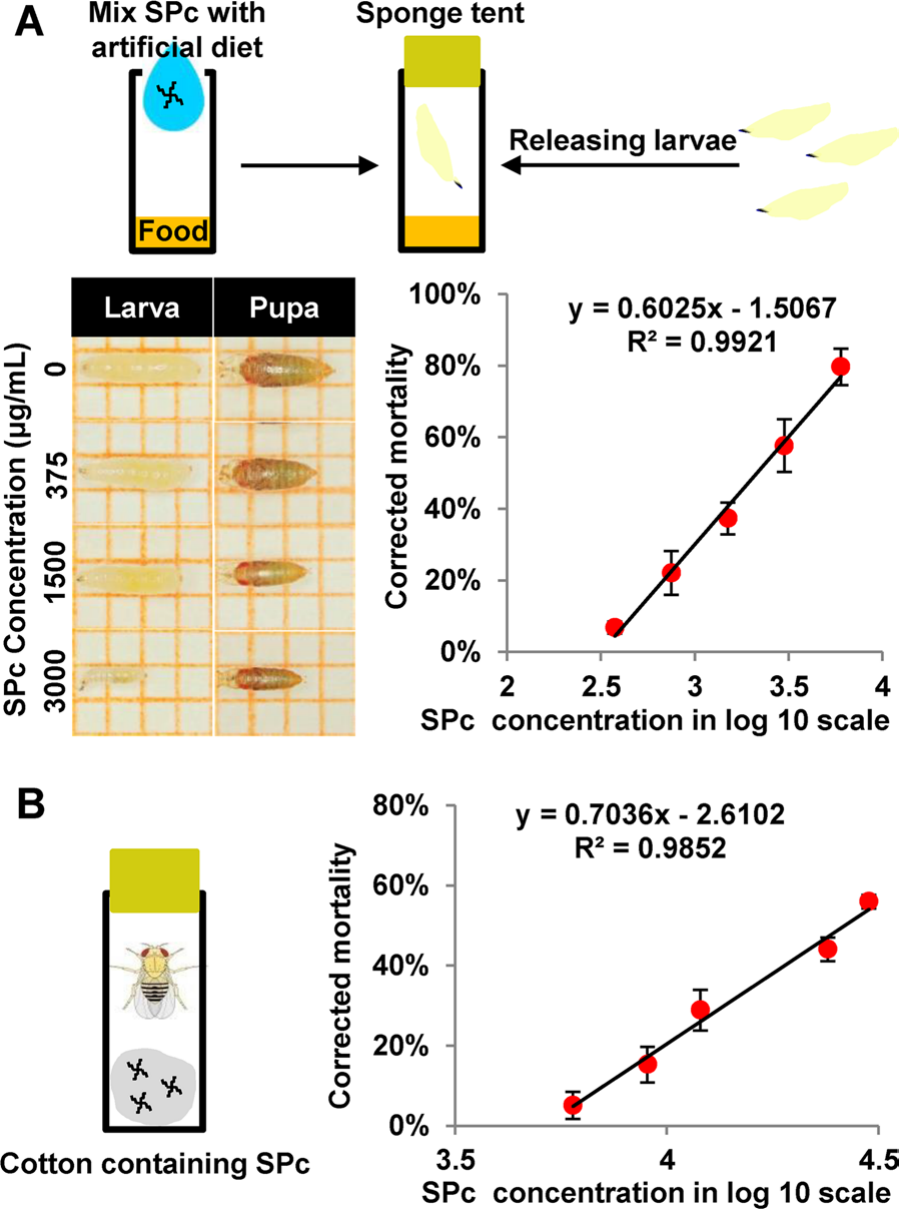
SPc causes lethality in fly larvae and adults. Various concentrations of SPc were used to determine the mortality rates over an interval of 24 h. The mortality rate and value of concentration in the log 10 scale were used to construct the toxicity regression equation for both larvae (**A**) and adult flies (**B**)

When fly larvae were exposed to various concentrations of SPc, lethality was observed at both larval and pupal stages (Fig. 2A). The mortality of third instar larvae was proportional to SPc concentration and the LC_50_ value was found to be 2.14 g/L (Fig. 2A). Feeding adult flies with SPc also led to acute lethality and the LC_50_ value was calculated to be 26.33 g/L (Fig. 2B).

### Chronic exposure to SPc in larval stage impairs adult life history traits

**Fig. 3:**
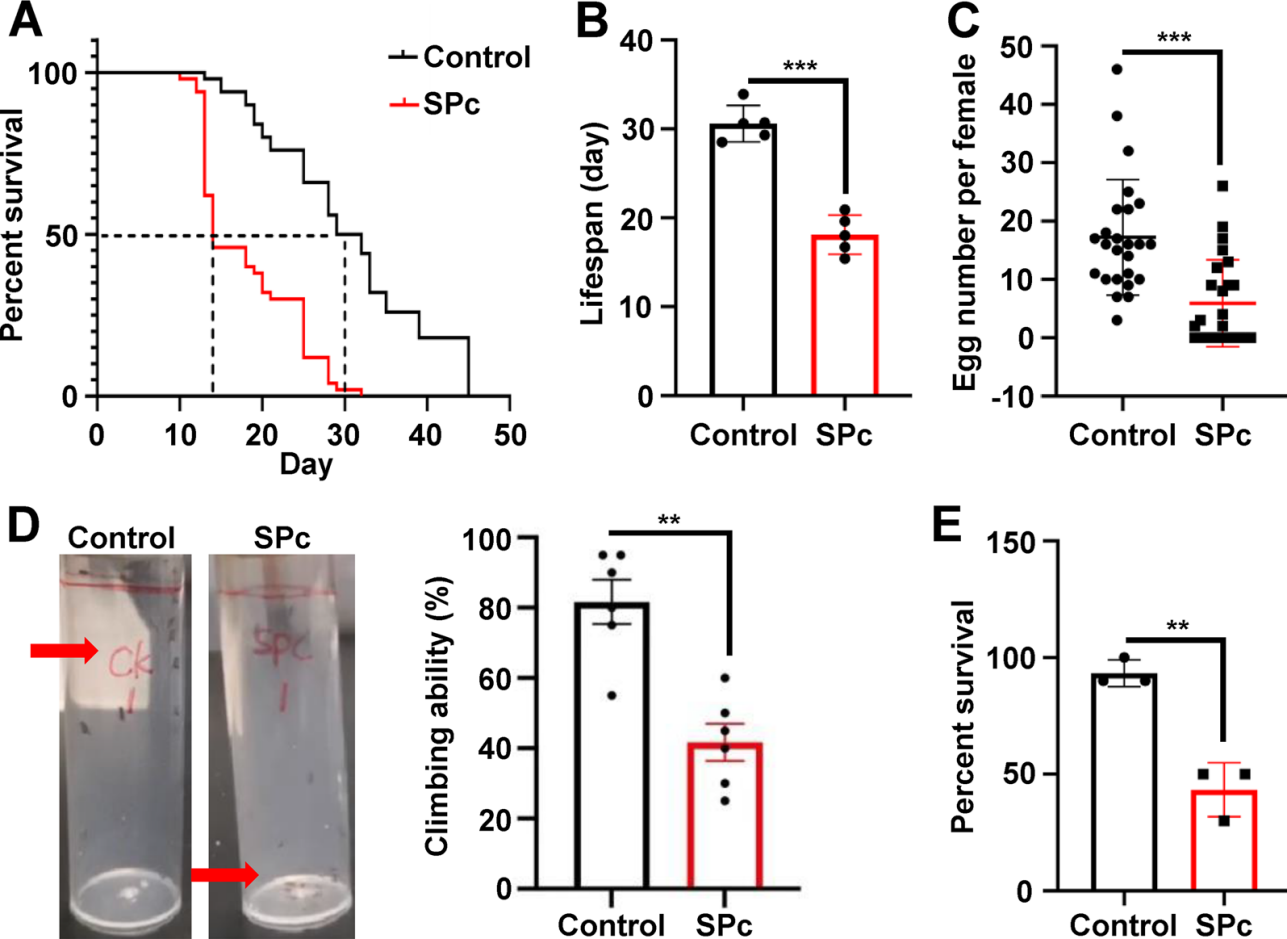
SPc treatment in larval stage results in dampened adult life history traits. **A** Effect of SPc on adult survival. **B** Effect of SPc on adult lifespan. **C** Effect of SPc on egg number. **D** Effect of SPc on adult climbing ability. **E** Effect of SPc on adult resistance to starvation and desiccation. The “**” and “***” indicate significant differences according to the independent *t* test (*P* < 0.01 and 0.001)

Whether chronic exposure to SPc in larval stage has long lasting effects was further examined. When first instar larvae were fed with food containing 1 g/L SPc, the emerged adults were morphologically indistinguishable with the control group, but their major life history traits were severely impaired. SPc exposure in larval stage reduced the median survival time from 30.5 days to 14 days in the emerged male adults (Fig. 3A), causing a 40.8% decline of lifespan which was reduced from 30.7 days to 16.7 days (Fig. 3B). After mating with normal male flies, the number of eggs laid per female was reduced from 17.2 to 5.9 after SPc treatment (Fig. 3C). Chronic exposure to SPc in larval stage resulted in 50% decline of climbing ability of emerged adults (Fig. 3D and Additional file: 2 and 3). When challenged with starvation and desiccation, larval stage SPc exposure impaired the survival of adults. Compared with the control group, SPc treatment decreased the resistance to starvation and desiccation by 53.5% (Fig. 3E).

### SPc induces prominent transcriptional responses in larvae

**Fig. 4:**
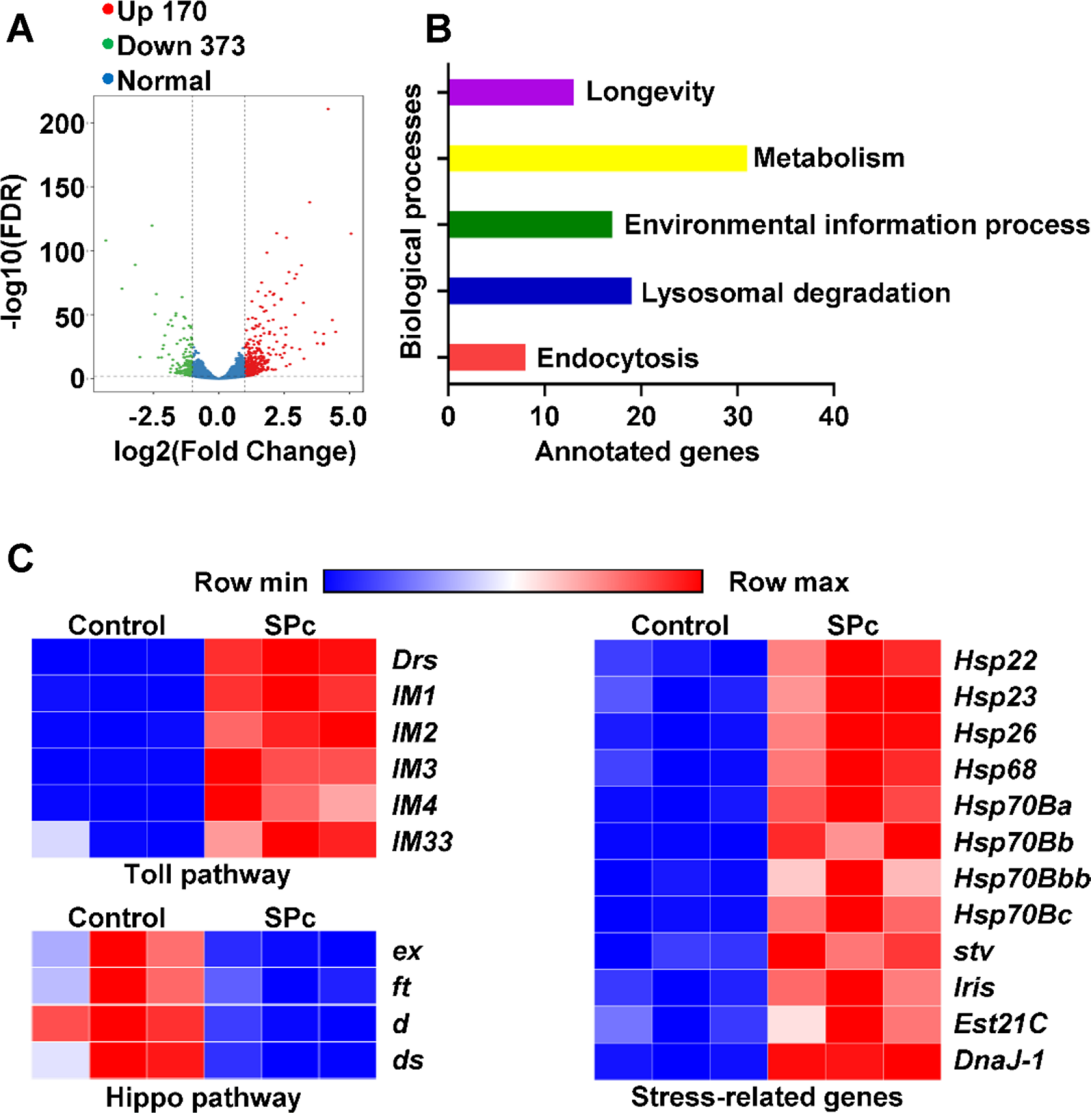
SPc induces measurable changes in larval gene expression. **A** Analysis of differentially expressed genes (DEGs) after feeding fly larvae with SPc nanoparticles shown as a volcano plot. Upregulated genes are represented by red dots and downregulated genes by green dots. **B** Analysis of biological processes affected by SPc. The X-axis is the number of DEGs, and the terms of biological processes are listed on the right side of each bar. **C** Heatmaps of the Toll signaling pathway, Hippo signaling pathway and stress response pathway. Highly expressed transcripts are labeled as red, while blue represents transcripts with low expression levels. Gene symbols are listed on the right side

To better understand the molecular mechanisms underlying the chronic toxicity of SPc nanoparticles, the change of gene expression profile upon SPc exposure in the larvae was investigated by transcriptomic analysis. SPc treatment resulted in 543 genes to be differentially expressed in fly larvae, among which 170 were up-regulated and 373 were down-regulated (Fig. 4A and Additional file 4: Table S1). SPc treatment led to profound effects on the following biological processes: endocytosis, lysosomal degradation, environmental information processing, metabolism and longevity (Fig. 4B). Further analysis revealed that SPc treatment strongly influenced the stress response pathways, Hippo signaling pathway, detoxification, ecdysone biosynthesis and Toll signaling mediated innate immunity pathway (Fig. 4C and Additional file 1: Fig. S2).

**Fig. 5:**
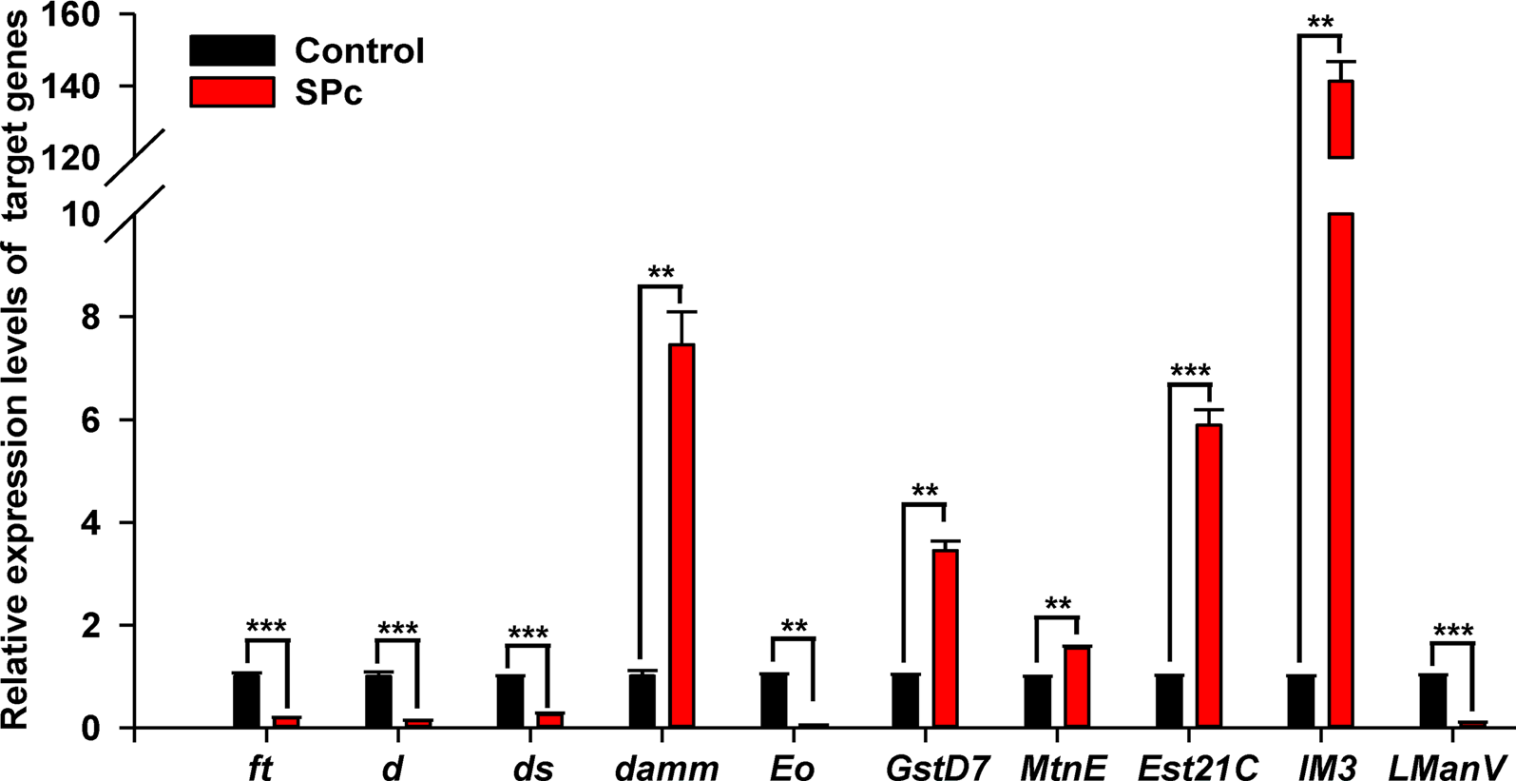
Fold-change in abundances of transcripts of *ft, d, ds, damm, Eo, GstD7, MtnE, Est21C, IM3* and *LManV* genes in response to SPc nanoparticles. The relative expression levels of target genes were normalized to the abundance of the *Rpl32* gene. The “**” and “***” indicate significant differences according to the independent *t* test (*P* < 0.01 and 0.001)

The qRT-PCR was used to validate the influence of SPc on mRNA levels of various genes (Fig. 5). The *ft, d* and *ds* genes encode essential components of the Hippo signaling pathway, and their mRNA levels were down-regulated by 0.20-fold, 0.14-fold and 0.26-fold, respectively. The apoptosis regulator gene *dam* was elevated by 7.46-fold when exposed to SPc. The ecdysone oxidase gene *Eo* was down-regulated by 0.05-fold. Expressions of antioxidant enzyme encoding gene *GstD7* was up-regulated by 3.45-fold. Metallothionein family gene *MtnE* was up-regulated by 1.56-fold. Stress response gene *Ets21C* was up-regulated by 5.90-fold. The innate immunity related gene *IM3* was strongly up-regulated by 141.22-fold. The lysosomal mannosidase gene *LManV* gene was down-regulated by 0.11-fold.

### SPc induces tissue specific responses

**Fig. 6:**
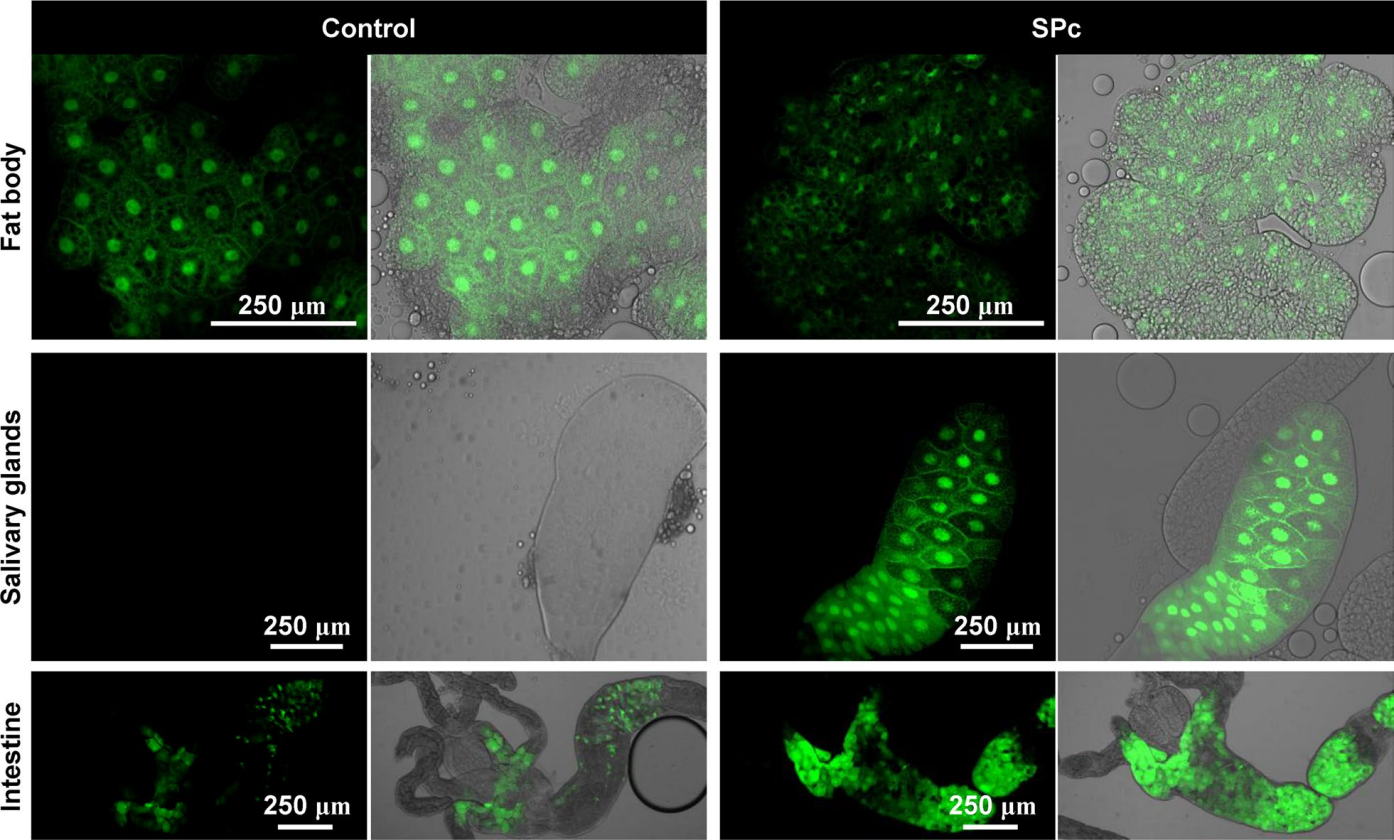
Effect of SPc on gstD-GFP expression in larval tissues. For each group, the fluorescence picture (green) and the merged picture of fluorescence and bright field panels were shown. Scale bar: 250 μm

**Fig. 7:**
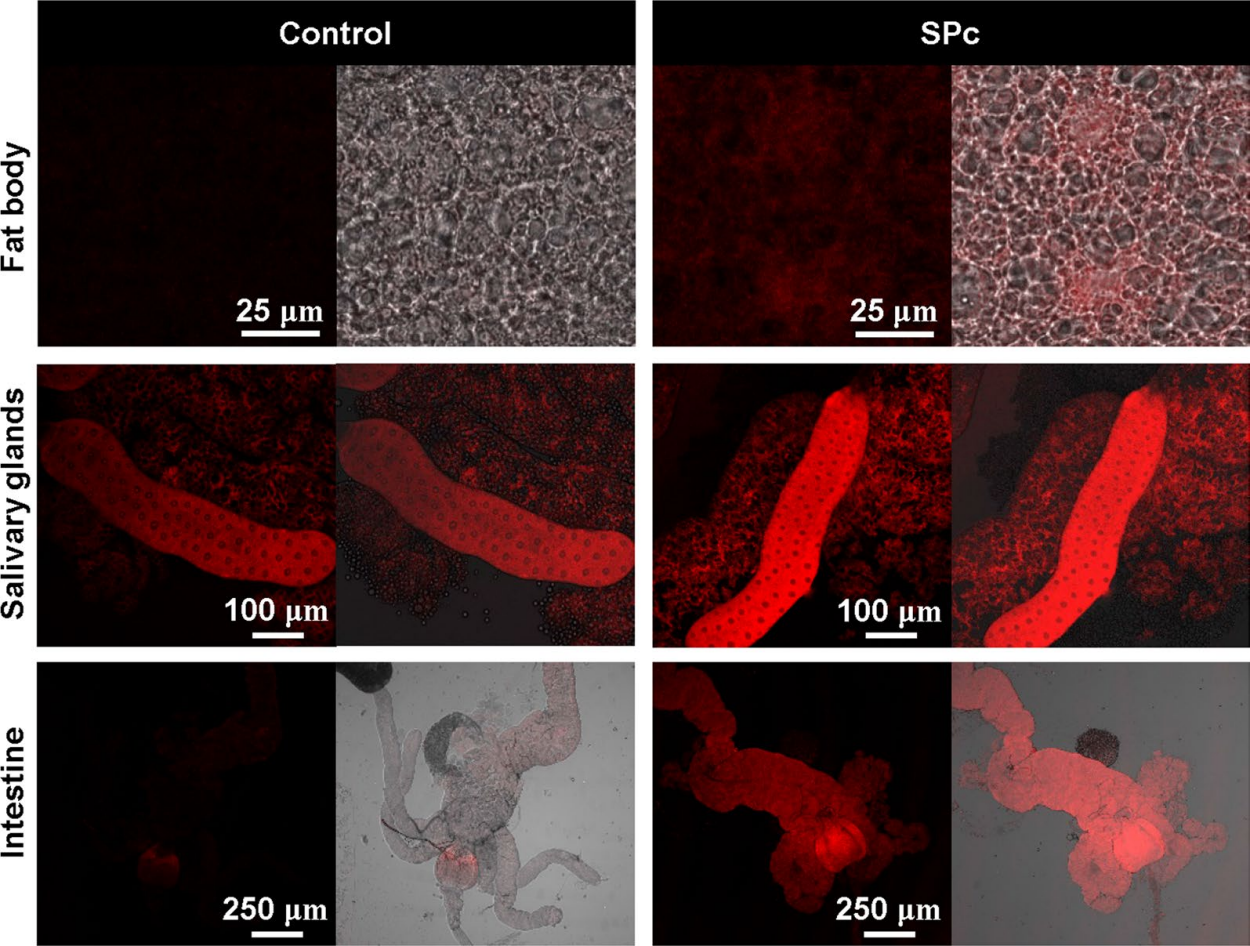
Effect of SPc on cellular ROS level in larval tissues. For each group, the fluorescence picture (red, DHE staining) and the merged picture of fluorescence and bright field panels were shown. Scale bars: 25 μm in fat body pictures; 100 μm in salivary gland pictures; 250 μm in intestine pictures

Expression of the gstD-GFP reporter [32] and cellular reactive oxygen species (ROS) level were examined upon SPc exposure. Feeding fly larvae with 1 g/L SPc led to significant change of gstD-GFP expression patterns in a tissue specific manner. Accumulation of gstD-GFP was found in the salivary gland and intestine, but not in the fat body (Fig. 6). Similar basal level of gstD-GFP expression was observed in the fat body of both control and SPc treated larvae. There was no detectable expression of gstD-GFP in the salivary glands of control larvae, while SPc stimulated the expression of gstD-GFP to a high level. Basal level of gstD-GFP expression was observed in the intestine of control larvae, but SPc treatment resulted in expansion of the expressing region as well as up-regulation of the expression level. Cellular ROS level was found to be elevated in fat body, salivary gland and intestine cells when examined by DHE staining (Fig. 7).

**Fig. 8:**
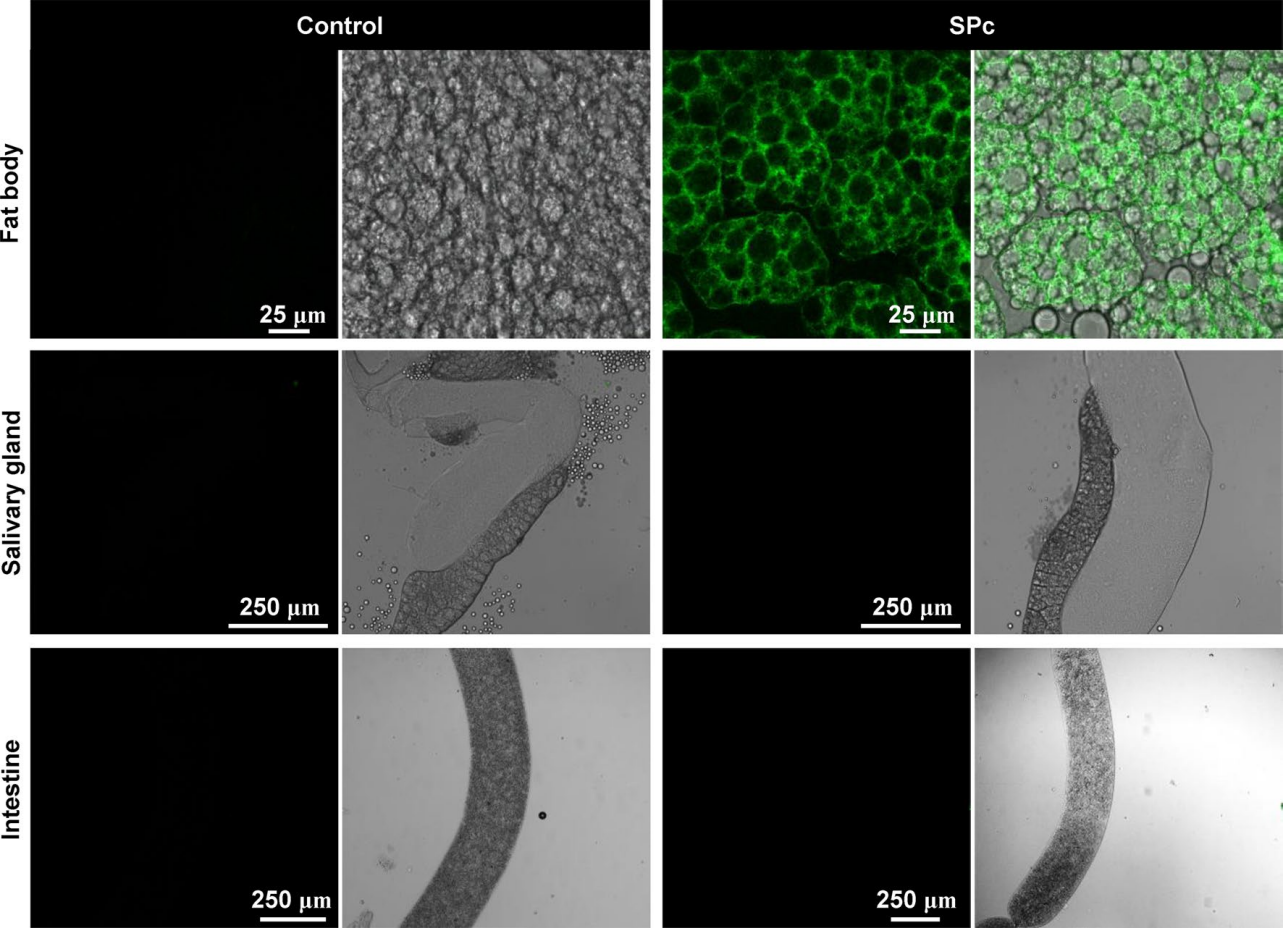
Effect of SPc on Toll signaling in larval tissues. For each group, the fluorescence picture (green) and the merged picture of fluorescence and bright field panels were shown. Scale bar: 25 μm in fat body pictures and 250 μm in salivary gland and intestine pictures

The impact of SPc nanoparticles on Toll signaling activity was further examined using the Drs-GFP reporter [33]. SPc nanoparticles were found to stimulate Drs-GFP expression in larvae fat body cells but not in the salivary gland and intestine cells (Fig. 8).

**Fig. 9:**
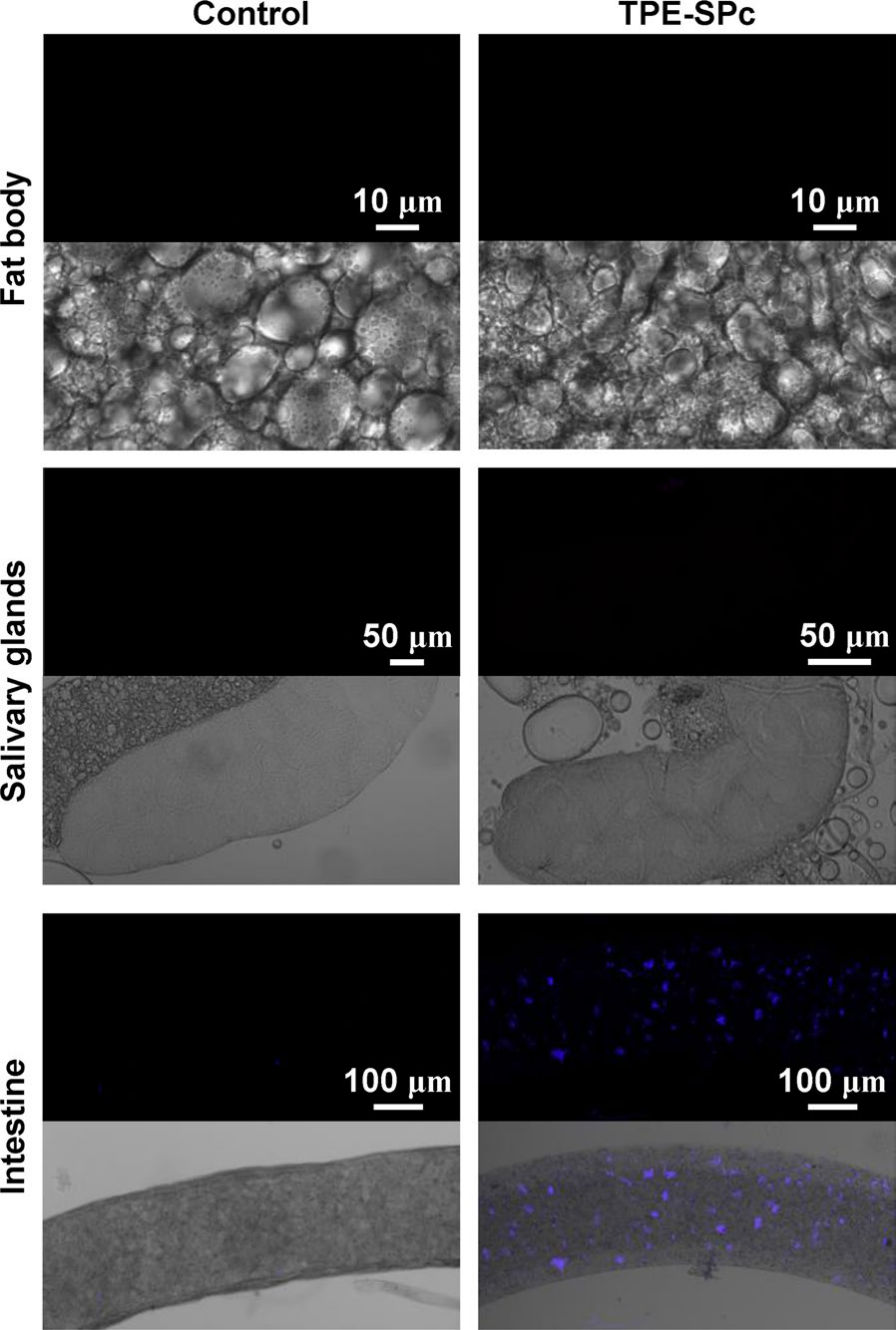
Distribution of SPc nanoparticles in larval tissues. For each group, the fluorescence picture (blue) and the merged picture of fluorescence and bright field panels were shown. Scale bars: 10 μm in fat body pictures; 50 μm in salivary gland pictures and 100 μm in intestine pictures

The tissue specific responses prompted us to examine whether SPc could be transported into different tissues after ingestion. Fluorescent-labelled SPc nanoparticles were synthesized and fed to fly larvae. Examination of SPc distribution in different tissues showed that SPc nanoparticles were mainly restricted in the intestine cells, with no visible traces in the salivary gland nor fat body cells (Fig. 9).

## Discussion

Using *Drosophila melanogaster* as the model organism, we showed that SPc nanoparticles were hazardous at multiple levels. In addition to the acute toxicity towards both fly larvae and adults, chronic exposure to SPc in the larval stage significantly impacted their life history traits. High-throughput RNA-seq analysis and subsequent qRT-PCR experiments demonstrated that SPc exposure could stimulate significant transcriptional changes in fly larvae. Functional classification of these genes would help us to better understand the toxicity of SPc at the molecular level.

Feeding *Drosophila* larvae with SPc induced up-regulation of genes involved in stress responses, including the heat shock proteins (HSPs). HSPs are molecular chaperons that play vital roles in reducing the harmful impacts when exposed in stressed conditions in nearly all living organisms [34]. Although named as heat shock proteins, HSPs could be induced by various stimulations such as cold shock, exposure to toxic compounds, desiccation, aging, oxidative stress and diseases [34, 35]. Both Stv [36, 37] and DNAJ-1 [38, 39] act as co-chaperone in the HSP chaperone machinery, while the expression of *Iris* also responds to fluctuation of temperature [40–42]. The *Ets21C* gene encodes another factor that regulates stress tolerance, tissue renewal and longevity [43, 44]. Induction of these stress response genes indicates that SPc nanoparticles might be recognized as hazardous xenobiotics by the fly larvae and could trigger a series of defensive mechanisms such as detoxification and immune response [45].

The GST (Glutathione S-transferases) and cytochrome P450 family of enzymes are well known for their roles of detoxification in insects [46, 47]. Upon ingestion of toxic substances, the expression level of *GST* and *P450* genes are highly up-regulated to meet the needs of converting hazardous substances into less toxic forms [48, 49]. Excessive amounts of heavy metals, especially the non-essential metals are detrimental for organisms [50]. Insects utilize the sulfyhydryl group-containing proteins metallothioneins (Mtns) to bind and sequester free metal ions to reduce the harmful impacts, and the expression of *Mtn* genes are efficiently induced by heavy metals [51–53]. The general induction of *GST*, *P450* and *Mtn* genes suggest that a strong and nonspecific detoxification response is triggered in fly larvae upon exposure to SPc nanoparticles.

Transcriptomic analysis indicated that expression of the genes encoding immune induced peptides, such as *IM1*, *IM2*, *IM3*, *IM4*, *IM33* and *Drs*, were up-regulated upon SPc exposure. Both the *IM* family genes and *Drs* are direct transcriptional targets of the Toll signaling pathway, which regulates innate immune response to pathogen-associated molecules [54–56]. The expression of *IM3* gene was up-regulated by 141.22-fold when examined by qRT-PCR, suggesting a strong activation of the Toll signaling. The induction of *Drs* was further visualized by a GFP reporter gene, which is expressed under the control of the *Drs* promoter and is able to faithfully reproduce the transcription pattern of the endogenous *Drs* gene [33]. SPc nanoparticles induced Drs-GFP in fly larval fat body cells but not in salivary gland nor intestine cells, which is consistent with the crucial role of fat body to produce antimicrobial peptides in systemic immune response [57].

SPc inhibited the expression of *Eo*, which is required for the production and release of a critical endocrine hormone known as ecdysone [58]. As ecdysone governs insect metamorphosis and reproduction [59], SPc may dampen fly development and female fertility through interfering ecdysone production. SPc also impacted the metabolism in fly larvae. LManV belongs to the class II α-mannosidases family which play important roles in the degradation of asparagine-linked carbohydrates of glycoproteins [60]. Deficiency of α-mannosidases causes the lysosomal storage disease [61], thus inhibition of *LManV* by SPc may lead to similar lysosomal storage defects which could also impair fly development and health. *Damm* encodes one of the caspase family of cysteine proteases whose overexpression induces cell death [62]. Induction of *Damm* expression suggests that cell death might be triggered by SPc. SPc treatment disrupted the expression of several components of the Hippo signaling pathway, which plays critical roles in both tissue development and innate immunity [63, 64].

Chronic exposure to SPc in the larval stage impairs adult life history traits, but the exact mechanisms are not fully understood. Upon SPc exposure, fly larvae experienced intense stresses which are known to compromise their overall fitness and impair major life history traits [65]. Prolonged immune activity is deleterious for growth and development [66, 67], which may contribute to the long-term effect of SPc. Metabolic abnormality and the hormonal changes in the larvae are related with impairments of adult lifespan and fecundity [68]. Further studies are required to reveal how SPc induce the long-lasting impacts.

The tissue specific responses upon SPc ingestion were further dissected by in vivo reporter assays. The transgenic gstD-GFP reporter was generated by fusing the regulatory sequences of the *GstD1* gene to GFP, and the resulting reporter is efficiently induced by oxidative stressors such as paraquat, arsenic or hydrogen peroxide [32]. The expression of gstD-GFP was induced by SPc in the intestine and salivary gland cells, while DHE staining demonstrated the cellular ROS level were also increased in these tissues. Collectively, these observations suggest that SPc might trigger a systemic oxidative stress. Activation of the immune response was limited to the fat body, as demonstrated by the Toll signaling activity reporter Drs-GFP. When distribution of SPc was tracked, only intestine cells were found to take in visible amounts of SPc nanoparticles. Although we could not rule out the possibility that very low level of SPc nanoparticles was transferred to other organs through the circulation system or by other means, we favor the model that SPc nanoparticles induce systemic responses after entering the intestine cells.

ROS was considered as a major cause of nanoparticle-induced toxicity in organisms [69–74]. But previous studies were focusing on the damages caused by ROS intracellularly [75–80]. Recently ROS were discovered to control cell differentiation and cellular immune response in a cell-non-autonomous fashion [81, 82]. We hypothesize that upon SPc exposure, ROS generated by the intestine cells serve as the messenger to induce oxidative stress and immune activity in other organs.

Recently, *Drosophila* has gained popularity as a model to study nanotoxicity [69–74, 83, 84]. The chronic toxicity of several types of nanocarriers have been tested in fly. When sub-lethal level of polylactic acid nanoparticles [85], cellulose nanofibrils [86] and lignin nanoparticles [87] were fed to fly larvae, the adult life history traits were weakly impaired (Additional file 5: Table S3). Cadmium oxide and silver nanoparticles caused similar impacts in the fly larvae and adults as SPc (Additional file 5: Table S3), while the underlying mechanisms awaits further exploration.

We have shown that the nanometerization of various pesticides by SPc results in enhanced toxicity against pests in both laboratory experiments and field tests [13–21, 88, 89]. SPc shows great potential as pesticide adjuvant for large-scale application in the crop fields, and our biotoxicity analysis could help to develop guidelines to reduce the environmental and health risks.

## Conclusions

Our experiments demonstrated that SPc are detrimental for *Drosophila* at multiple levels. Chronic exposure to SPc at sublethal level concentration showed long lasting adverse effects on longevity, reproduction and motor activity. Genes and signaling pathways related with these defects were identified and systemic responses were observed. These results provide reference for understanding the hazards of SPc nanocarriers and for developing guidelines for large scale applications in the crop field.

## Methods

### Synthesis of SPc

SPc was synthesized following a previously described method [12]. Briefly, the star initiator Pt-Br is constructed by adding 2-bromo-2-methylpropionyl bromide (HEOWNS) dropwise into the pentaerythritol (Alfa Aesar) solution in dry tetrahydrofuran and triethylamine (Beijing Chemical Works). The initiator Pt-Br was further polymerized with DMAEMA (Energy Chemical) under a nitrogen atmosphere with the help of tetrahydrofuran, PMDETA (Sigma-Aldrich) and CuBr (Sigma-Aldrich). Dialysis was carried out to purify the crude product and SPc was obtained as white powder after being freeze-dried. A 60 g/L stock of SPc was prepared with double distilled water (ddH_2_O). To synthesize fluorescent SPc, TPE-pentaerythritol was used instead of pentaerythritol to prepare the TPE-4Br star initiator and the subsequent steps were the same.

### Particle size and zeta potential measurement

Samples of SPc were diluted with ddH_2_O to prepare 0.1 mg/mL solution, which were used for measurement of particle size by scanning electron microscope (JSM-7500F, JEOL Ltd.) and of zeta potential by Zetasizer Nano ZS (Malvern Instruments Ltd., UK). Each assay was repeated 3 times at 25 °C.

### Drosophila culture

The *Canton-S* (Bloomington 64349) *Drosophila* strain was used to evaluate the toxicity of SPc. The fly stocks were reared with standard fly food and cultured in a constant temperature incubator at 25 °C [88]. The fly food was composed of corn meal, agar, sucrose, glucose, yeast, propionic acid and the anti-fungal agent Tegosept (p-hydroxybenzoic acid, methyl ester, methyl paraben, nipage). The gstD-GFP [32] stock was used to visualize oxidation stress in the larvae. The Drs-GFP (Bloomington 55707) stock was used to visualize the Toll pathway regulated immune activity in the larvae.

### Acute toxicity assay

Acute toxicity of SPc was examined by feeding *Drosophila* larvae with food containing SPc nanoparticles. Fly food were prepared with different concentrations of SPc (6, 3, 1.5, 0.75, 0.375 and 0 g/L) and twenty third instar larvae were transferred into each vial. Adult flies were fed with 1% sucrose solutions containing different concentrations of SPc (30, 24, 12, 9, 6 and 0 g/L), for which purpose cotton wads soaked with sucrose solutions along with twenty newly emerged adult flies were put into each vial. Three replicates were carried out for each SPc concentration in both larvae and adult toxicity experiments. The number of deaths in each vial was recorded after 24 h and the LC_50_ value of SPc was calculated through probit analysis [90].

### Effects of SPc nanoparticles at sub-lethal concentration on D. melanogaster

In order to determine the potential adverse effects of chronical exposure to SPc nanoparticles at sub-lethal concentration, first instar larvae were exposed for 7 days to 1 g/L SPc nanoparticles (equivalent to the value of LC_30_). The SPc nanoparticles were supplemented in the fly food. The emerged adult flies were transferred onto fresh fly food without SPc nanoparticles, and their lifespan, fecundity, climbing ability and stress resistance were examined. The gene expression changes were also examined in third instar larvae.

### Examination of adult lifespan

Lifespan assay was conducted following previously described methods [91, 92]. A total of 10 adult flies were cultured in each vial and 5 vials were examined simultaneously for both control and SPc treatment group. Food was changed every 5 days, and the number of deaths was recorded each day till the death of the last fly. To avoid complications caused by differences between the sexes and ages, one day old males were used in the lifespan assay.

### Examination of female fecundity

Virgin female flies were picked from both control and SPc treatment group. Single virgin female fly was crossed with three wild type males for 24 h and the fecundity was measured by counting the number of eggs laid by each female fly. Two days old virgin flies were used for the fecundity assay.

### Examination of adult climbing ability

Adult fly climbing ability was examined as a marker for their motor activity [25, 26]. A total of 20 adult flies were put into an empty vial and 5 vials were examined simultaneously for both control and SPc treatment group. The flies were gently tapped to the bottom, and upward movement of flies was videotaped for 30 s. The number of flies that climbed 20 cm height (marked by red line on the vial) in 30 s were counted. Five days old flies were used in the climbing assay.

### Examination of adult resistance

Adult resistance to starvation and desiccation was tested by putting ten adults into an empty vial. The number of deaths was recorded after 12 h. Three vials were examined simultaneously for both control and SPc treatment group. Five days old flies were used in the resistance assay.

### Transcriptomic analysis for gene expression changes

Total RNAs were isolated from control and SPc nanoparticles exposed third instar larvae using RNA simple Total RNA Kit (Tiangen, Beijing, China). The RNA sequencing libraries were constructed and then sequenced using an Illumina Hiseq platform (Biomics, Beijing, China). Analyzed by the DESeq2 R package, genes with fold change ≥ 2 between the control and SPc treatment group and with false discovery rate < 0.01 were considered to be differentially expressed. The list of differentially expressed genes was provided in Additional file 4: Table S1.

### Quantitative RT-PCR (qRT-PCR)

Total RNAs were extracted from control and SPc nanoparticles exposed third instar larvae using TRIeasy (Yeasen Biotech, Shanghai, China). The cDNAs were synthesized by the Hifair First Strand cDNA Synthesis Kit (Yeasen Biotech) and used as templates for PCR experiments using the Perfect Start Green qPCR Super Mix (TransGen Biotech, Beijing, China). qRT-PCR were conducted on an ABI QuantStudio 6 Flex System (Thermo Fisher, USA). The *Rpl32* gene was used as internal control for qRT-PCR, and the gene expression level was examined by the ΔΔCt method [88, 92]. The primers used for qRT-PCR in this study are listed in Additional file 6: Table S2.

### Tissue and fluorescent imaging

The expression of gstD-GFP and Drs-GFP as well as the distribution of TE-SPc nanoparticles were monitored under a Leica SP8 confocal microscope. The cellular ROS level was examined by DHE staining [93]. Fluorescent photos were captured with Leica SP8 confocal microscope. The laser intensity and exposure time was set at the same value when samples from the control group and SPc treatment group were photographed.

### Data analysis

The statistical analysis was performed using the SPSS 19.0 software (SPSS Inc., USA). The data was analyzed using the one-way ANOVA with the Tukey HSD test or independent *t*-test with the *P*-value < 0.05 recognized as significant difference. The descriptive statistics are shown as the mean value and standard errors of the mean.

## Supplementary Information

**Additional file 1: Fig. S1.** SEM images of SPc nanoparticles. SPc nanoparticles are found to be spherical in shape at both concentrations. **Fig. S2.** Heatmaps of the detoxification genes, lysosome related genes and ecdysone biosynthesis genes. Highly expressed transcripts are labeled as red, while blue represents transcripts with low expression levels. Gene symbols are listed on the right side.

**Additional file 2: Mov. S1.** Representive video of the climbing ability test using wild type flies.

**Additional file 3: Mov. S2.** Representive video of the climbing ability test using SPc treated flies.

**Additional file 4: Table S1.** List of differentially expressed genes upon SPc treatment.

**Additional file 5: Table S3.** Chronic toxicity of nanomaterials as tested in the fruit fly.

**Additional file 6: Table S2.** Primers for quantitative real-time PCR.

## Data Availability

All data generated or analysed during this study are included in this published article [and its supplementary information files].
