# Supplementary material for: Chronic exposure to the star polycation (SPc) nanocarrier in the larval stage adversely impairs life history traits in Drosophila melanogaster
Source: J Nanobiotechnology. 2022 Dec 8;20:515. doi: 10.1186/s12951-022-01705-1 (PMC9730587; doi:10.1186/s12951-022-01705-1)
Supplement: Supplementary file 1 — Additional file 1: Fig. S1. SEM images of SPc nanoparticles. SPc nanoparticles are found to be spherical in shape at both concentrations. Fig. S2. Heatmaps of the detoxification genes, lysosome related genes and ecdysone biosynthesis genes. Highly expressed transcripts are labeled as red, while blue represents transcripts with low expression levels. Gene symbols are listed on the right side. [file 12951_2022_1705_MOESM1_ESM.docx]

Supplementary Figure Legends


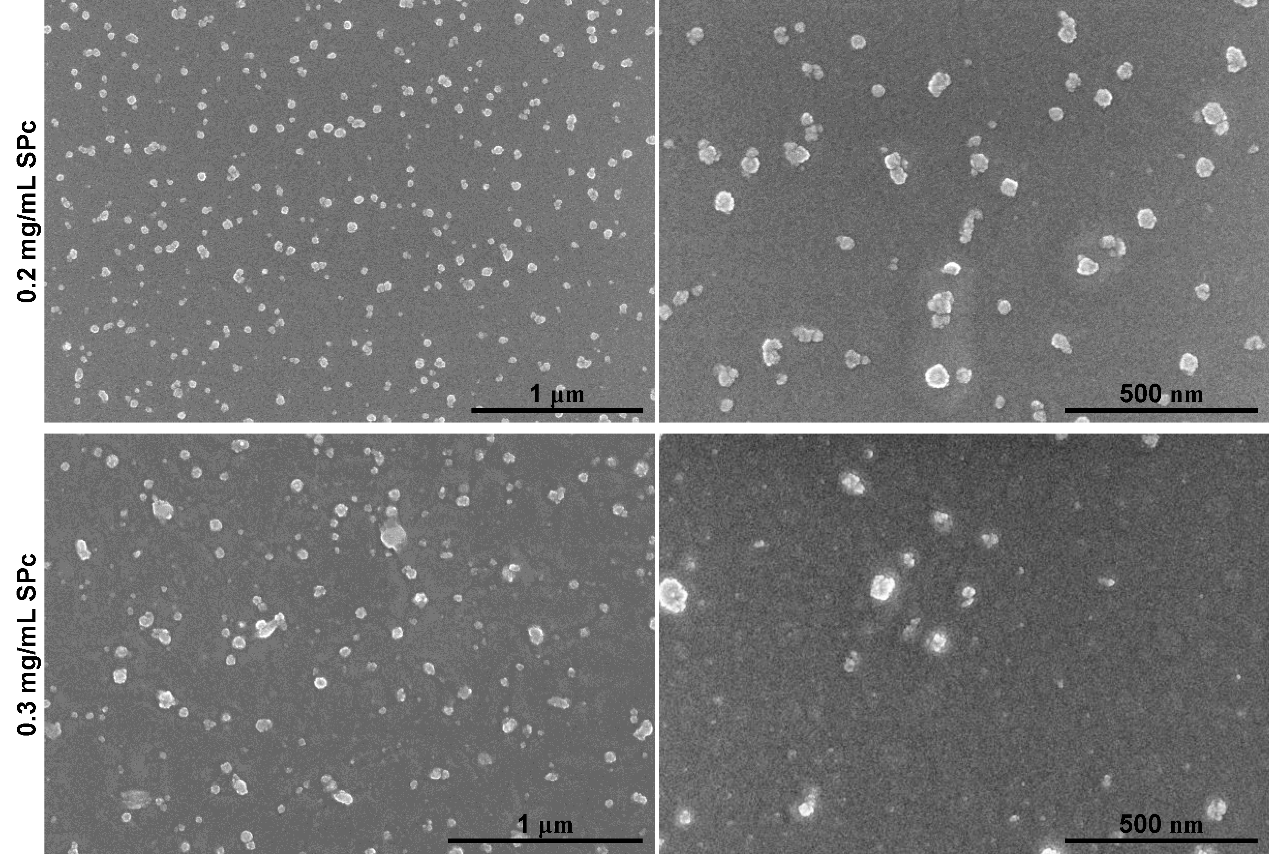


Fig. S1. SEM images of SPc nanoparticles. SPc nanoparticles are found to be spherical in shape at both concentrations.

.


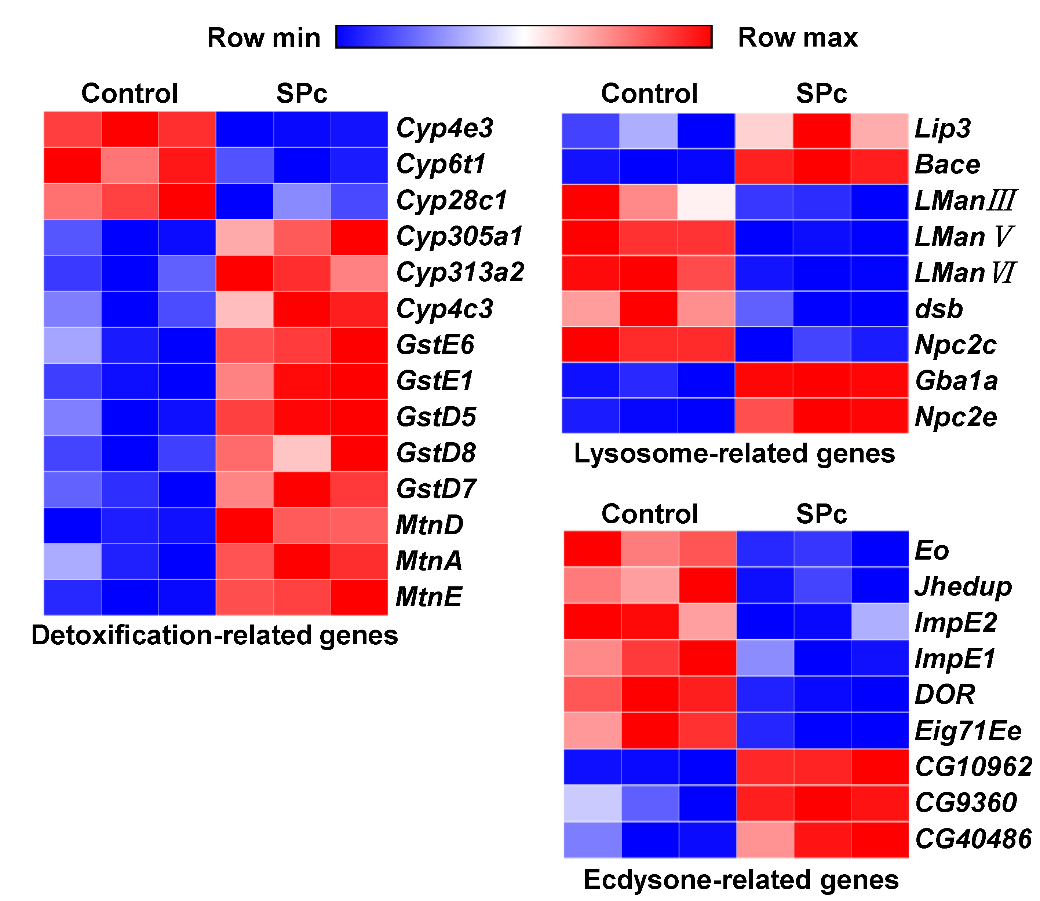


Fig. S2. Heatmaps of the detoxification genes, lysosome related genes and ecdysone biosynthesis genes. Highly expressed transcripts are labeled as red, while blue represents transcripts with low expression levels. Gene symbols are listed on the right side.
