# Supplementary material for: Chronic exposure to the star polycation (SPc) nanocarrier in the larval stage adversely impairs life history traits in Drosophila melanogaster
Source: J Nanobiotechnology. 2022 Dec 8;20:515. doi: 10.1186/s12951-022-01705-1 (PMC9730587; doi:10.1186/s12951-022-01705-1)
Supplement: Supplementary file 5 — Additional file 5: Table S3. Chronic toxicity of nanomaterials as tested in the fruit fly. [file 12951_2022_1705_MOESM5_ESM.docx]

**Table S3 Chronic toxicity of nanomaterials as tested in the fruit fly**

| Nanomaterial | Stress Response | Detoxification | Immune  Response | ROS and oxidative stress | Lifespan | Fecundity | Motor activity | Genotoxicity | Other Effects | Reference |
| --- | --- | --- | --- | --- | --- | --- | --- | --- | --- | --- |
| SPc | Yes | Yes | Yes | Yes | Reduced | Reduced | Reduced | Not tested |  | This study |
| Cadmium oxide (CdO) | Not tested | Mtn genes up-regulated | Not tested | CAT and SOD up-regulated | Reduced | Reduced | Reduced | Not tested | Blistered wing | El Kholy et al., 2021 |
| Aluminum oxide (Al_2_O_3_) | Not tested | Not tested | Not tested | Not tested | Not tested | Not tested | Reduced | No | Loss of appendages | Demir et al., 2013; Anand et al., 2019 |
| Zinc oxide (ZnO) | Hsp70 down-regulated | Not tested | Not tested | Yes | No significant effect | Not tested | Reduced | Weak | Wing deformation in the progeny | Alaraby et al., 2015; Carmona et al., 2016; Anand et al., 2017 |
| Titanium dioxide (TiO2) | Yes | Not tested | Not tested | Yes | No effect or not tested | Not tested | Not tested | Yes |  | Posgai et al., 2011; Demir et al., 2013; Demir, 2020; Alaraby et al., 2021 |
| Silica dioxide (SiO2) | Yes | Not tested | Not tested | Yes | Not tested | Not tested | Not tested | DNA damage in the hemocytes, no effect in the wing spot test |  | Pandey et al., 2013; Demir et al., 2015 |
| Silver (Ag) Nanoparticles | Yes | Yes | Yes | Yes | Reduced | Reduced | Reduced | Yes | Reduction in body pigmentation | Ahamed et al., 2010; Panacek et al., 2011; Mao et al., 2018; Wang et al., 2023 |
| Polylactic acid nanoparticles | Not tested | Not tested | Not tested | Not tested | No effect | Not tested | Not tested | Not tested |  | Legaz et al., 2016 |
| Cellulose nanofibrils | Not tested | Not tested | Not tested | Reduced | Not tested | Not tested | Reduced | Not tested | Adult weight decreased | Mishra et al., 2019 |
| Lignin nanoparticles | Not tested | Yes | Not tested | Not tested | Not tested | Not tested | Reduced | No |  | Siddiqui et al., 2020 |

**References**

El Kholy S, Giesy JP, Al Naggar Y. 2021. Consequences of a short-term exposure to a sub lethal concentration of CdO nanoparticles on key life history traits in the fruit fly (Drosophila melanogaster). *J Hazard Mater*. 410:124671.

Anand AS, Gahlot U, Prasad DN, Kohli E. 2019. Aluminum oxide nanoparticles mediated toxicity, loss of appendages in progeny of Drosophila melanogaster on chronic exposure. *Nanotoxicology*. 13:977-989.

Alaraby M, Annangi B, Hernández A, Creus A, Marcos R. 2015. A comprehensive study of the harmful effects of ZnO nanoparticles using Drosophila melanogaster as an in vivo model. *J Hazard Mater*. 296:166-174.

Anand AS, Prasad DN, Singh SB, Kohli E. 2017. Chronic exposure of zinc oxide nanoparticles causes deviant phenotype in Drosophila melanogaster. *J Hazard Mater*. 327:180-186.

Posgai R, Cipolla-McCulloch CB, Murphy KR, Hussain SM, Rowe JJ, Nielsen MG. 2011. Differential toxicity of silver and titanium dioxide nanoparticles on Drosophila melanogaster development, reproductive effort, and viability: size, coatings and antioxidants matter. *Chemosphere*. 85:34-42.

Demir E, Aksakal S, Turna F, Kaya B, Marcos R. 2015. In vivo genotoxic effects of four different nano-sizes forms of silica nanoparticles in Drosophila melanogaster. *J Hazard Mater*.283:260-266.

Panacek A, Prucek R, Safarova D, Dittrich M, Richtrova J, Benickova K, Zboril R, Kvitek L. 2011. Acute and chronic toxicity effects of silver nanoparticles (NPs) on Drosophila melanogaster. *Environ Sci Technol.* 45:4974-4979.
