# Supplementary material for: Chronic exposure to the star polycation (SPc) nanocarrier in the larval stage adversely impairs life history traits in Drosophila melanogaster
Source: J Nanobiotechnology. 2022 Dec 8;20:515. doi: 10.1186/s12951-022-01705-1 (PMC9730587; doi:10.1186/s12951-022-01705-1)
Supplement: Supplementary file 6 — Additional file 6: Table S2. Primers for quantitative real-time PCR. [file 12951_2022_1705_MOESM6_ESM.docx]

**Table S2. Primers for quantitative real-time PCR.**

| Gene | Primer | Sequence |
| --- | --- | --- |
|  | Ef-1α-R | GCGTGTTCACGAGTTTGTCC |
| *ft* | ft-F | GGTGGTGAGACGAGCAGAAA |
|  | ft-R | TCGAATCCATCGTAGGCGTG |
| *d* | d-F | GAAACGATCGCACAAAACCCA |
|  | d-R | CGAATTTGCTGGTGGCACTT |
| *ds* | ds-F | ACATTATTGTGGCGGAGGCA |
|  | ds-R | CCAGTTTGACCTTTGTGCGG |
| *Damm* | Damm-F | AATCAATCGAAGCGGAACCC |
|  | Damm-R | GCGCGTTATAATGCCTACCC |
| *Eo* | Eo-F | GCTCAAGTTCAGCGGGTACT |
|  | Eo-R | CCTAACGTTTTCGCCGTGTG |
| *GstD7* | GstD7-F | GGTGACCAACTGAAGCCAGA |
|  | GstD7-R | CGCGACTCCCAGATGACAAA |
| *MtnE* | MtnE-F | ATGCCTTGCAAGGGATGTGG |
|  | MtnE-R | TCACTTGGCCTGGCAGCACT |
| *Ets21C* | Ets21C-F | CGGATCCGGAAACGAGACC |
|  | Ets21C-R | CCGGAATCCGAGTCTGTTGA |
| *IM3* | IM3-F | TCACTCGCCTTCGTTTTGGG |
|  | IM3-R | TTAGGCCCTCACATTGCAGAC |
| *LManV* | LManV-F | GCCATCACTGGAACCGAGAA |
|  | LManV-R | GCAGGCAACTCTCGAACTCT |
| *Rpl32* | RPL32-F | ATGCTAAGCTGTCGCACAAATG |
|  | RPL32-R | GTTCGATCCGTAACCGATGT |
